# Characterization of *Legionella* Species from Watersheds in British Columbia, Canada

**DOI:** 10.1128/mSphere.00246-17

**Published:** 2017-08-02

**Authors:** Michael A. Peabody, Jason A. Caravas, Shatavia S. Morrison, Jeffrey W. Mercante, Natalie A. Prystajecky, Brian H. Raphael, Fiona S. L. Brinkman

**Affiliations:** a Department of Molecular Biology and Biochemistry, Simon Fraser University, Burnaby, British Columbia, Canada; b Respiratory Diseases Branch, Centers for Disease Control and Prevention, Atlanta, Georgia, USA; c Department of Pathology and Laboratory Medicine, University of British Columbia, Vancouver, British Columbia, Canada; d British Columbia Centre for Disease Control Public Health Laboratory, Vancouver, British Columbia, Canada; University of Wisconsin—Madison

**Keywords:** Legionella, metagenomics, watersheds

## Abstract

*Legionella* spp. present in some human-made water systems can cause Legionnaires’ disease in susceptible individuals. Although legionellae have been isolated from the natural environment, variations in the organism’s abundance over time and its relationship to aquatic microbiota are poorly understood. Here, we investigated the presence and diversity of legionellae through 16S rRNA gene amplicon and metagenomic sequencing of DNA from isolates collected from seven sites in three watersheds with varied land uses over a period of 1 year. *Legionella* spp. were found in all watersheds and sampling sites, comprising up to 2.1% of the bacterial community composition. The relative abundance of *Legionella* tended to be higher in pristine sites than in sites affected by agricultural activity. The relative abundance levels of *Amoebozoa*, some of which are natural hosts of legionellae, were similarly higher in pristine sites. Compared to other bacterial genera detected, *Legionella* had both the highest richness and highest alpha diversity. Our findings indicate that a highly diverse population of legionellae may be found in a variety of natural aquatic sources. Further characterization of these diverse natural populations of *Legionella* will help inform prevention and control efforts aimed at reducing the risk of *Legionella* colonization of built environments, which could ultimately decrease the risk of human disease.

**IMPORTANCE** Many species of *Legionella* can cause Legionnaires’ disease, a significant cause of bacterial pneumonia. *Legionella* in human-made water systems such as cooling towers and building plumbing systems are the primary sources of Legionnaires’ disease outbreaks. In this temporal study of natural aquatic environments, *Legionella* relative abundance was shown to vary in watersheds associated with different land uses. Analysis of the *Legionella* sequences detected at these sites revealed highly diverse populations that included potentially novel *Legionella* species. These findings have important implications for understanding the ecology of *Legionella* and control measures for this pathogen that are aimed at reducing human disease.

## Abstract (precis)

Many species of *Legionella* can cause Legionnaires’ disease, a significant cause of bacterial pneumonia. *Legionella* in human-made water systems such as cooling towers and building plumbing systems are the primary sources of Legionnaires’ disease outbreaks. In this temporal study of natural aquatic environments, *Legionella* relative abundance was shown to vary in watersheds associated with different land uses. Analysis of the *Legionella* sequences detected at these sites revealed highly diverse populations that included potentially novel *Legionella* species. These findings have important implications for understanding the ecology of *Legionella* and control measures for this pathogen that are aimed at reducing human disease.

## INTRODUCTION

Legionnaires’ disease (LD) is a potentially fatal form of bacterial pneumonia caused by various species of *Legionella* (1). Individuals with chronic lung diseases, immune system deficiencies, smokers, and people of advanced age are at an increased risk for LD. A milder form of legionellosis characterized by fever and “flu-like” symptoms is termed Pontiac fever. In built environments, *Legionella* can multiply in water that is stagnant, maintained at permissive temperatures (~25 to 37°C), and that lacks appropriate disinfectant levels. Various devices, such as cooling towers, showerheads, fountains, and spas, can aerosolize contaminated water. Inhalation of these aerosols by susceptible individuals can result in legionellosis.

Over 60 species of *Legionella* have been identified (http://www.bacterio.net/-allnamesdl.html) and at least one-third of these have been linked to human disease (2). *L. pneumophila* is the most frequent cause of LD in North America. Other less common, clinically relevant species include *L. longbeachae*, *L. bozemanii*, *L. micdadei*, and *L. dumoffii* (3–6) *L. pneumophila* is highly diverse, with 17 known serogroups (1). Genome sequence analysis has revealed that much of the genetic diversity among isolates of *L. pneumophila* is driven by recombination (7).

During legionellosis outbreak investigations, *Legionella* isolates from potential environmental sources are compared with clinical isolates in an effort to support epidemiological associations. Various subtyping schemes have been used for this purpose, such as pulsed-field gel electrophoresis, sequence-based typing, and more recently, whole-genome sequencing (1, 8). Confirmation of the environmental sources of *Legionella* may help to shorten the duration of an outbreak by focusing remediation efforts on a specific source and by informing ongoing prevention strategies.

The mechanisms by which sources of *Legionella* from the natural environment colonize the built environment are poorly understood. However, it is likely that at least some of the *Legionella* strains present in source water for built environments is derived from natural aquatic ecosystems, such as rivers, streams, and lakes, where *Legionella* strains have been shown to be widely distributed (9, 10). Various studies have demonstrated a link between *Legionella* and various protozoa, including amoebae such *Acanthamoeba* spp., *Naegleria* spp., and *Hartmannella* spp. (11, 12). Many of the molecular mechanisms that legionellae use for growth in amoebae appear to overlap those for growth in human macrophages (13). Moreover, growth within amoebae not only amplifies the number of *Legionella* organisms but also may enhance bacterial virulence (13, 14). Finally, *Legionella* spp. have been detected in biofilms, which are considered a major reservoir of the organism in colonized human-made water systems (15, 16).

Few studies have attempted a comprehensive analysis of the microbiome of natural aquatic environments. Recently, a year-long study was conducted to understand the microbial community composition in various watersheds with different land uses in British Columbia, Canada (17, 18). In this study, sites within agricultural, urban, and protected watersheds were sampled monthly. Size fractionation methods were employed to generate templates for sequencing. 16S and 18S amplicon sequencing was conducted to quantify changes in the microbiomes of these environments, while shotgun metagenomic sequencing was conducted to understand community structure and function. Notably, this study revealed that the most abundant bacterial phyla present in the watersheds studied were *Proteobacteria* (which includes *Legionella*), *Actinobacteria*, *Firmicutes*, and *Bacteroidetes* (17).

In order to better understand the ecology of *Legionella* in the natural aquatic environment, we evaluated this extensive data set with the goals of quantifying *Legionella* abundance among different watersheds, determining the diversity of *Legionella* spp. present, and evaluating the role of amoeba when in the presence of this bacterium in such environments. Understanding the presence and diversity of *Legionella* in these watersheds may help to improve our ability to control colonization by these organisms of human-made water systems from natural water sources.

## RESULTS

### Detection of *Legionella* over time at various sampling sites.

**FIG 1:**
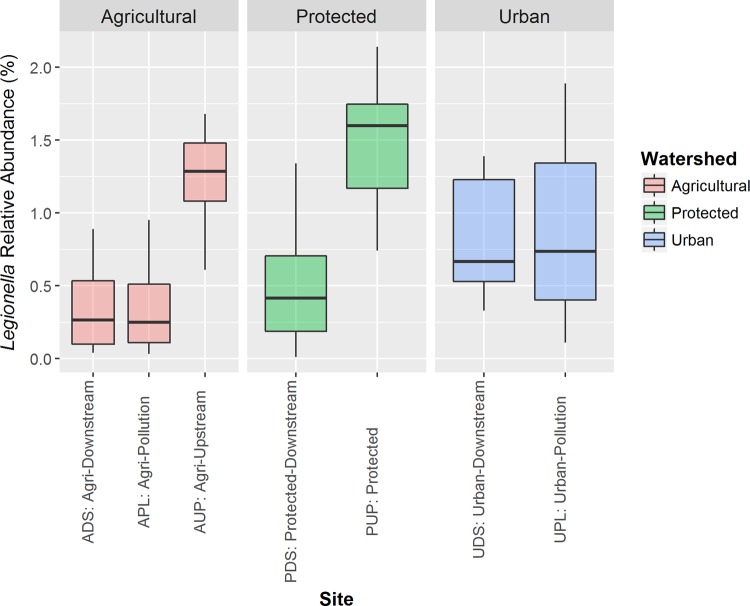
Abundance of *Legionella* species at various sites. The box plots represent the relative abundances of *Legionella* present for all samples obtained from the indicated site, which was determined by 16S rRNA sequencing. Boxes are bounded by 25th and 75th percentiles, and the middle line represents the median relative abundance.

Analysis of the 16S data set revealed that *Legionella* spp. were found in all watersheds and sampling sites (Fig. 1). Overall, the relative abundances of *Legionella* spp. were ≤2.1% of the bacterial taxa present and were significantly higher (*q*-value, <0.05) (see Table S1 in our supplementary files posted on figshare [https://doi.org/10.6084/m9.figshare.5046937]) in samples from more-pristine sites (protected upstream [PUP] samples and upstream of agricultural activity [AUP] samples) than from sites affected by agricultural activity, while the sites affected by urban activity had intermediate relative abundance levels of *Legionella* spp. The relative abundance of *Legionella* spp. varied substantially over the sampling period (see Fig. S1).

### Distribution of *Legionella* species among sampling sites.

**FIG 2:**
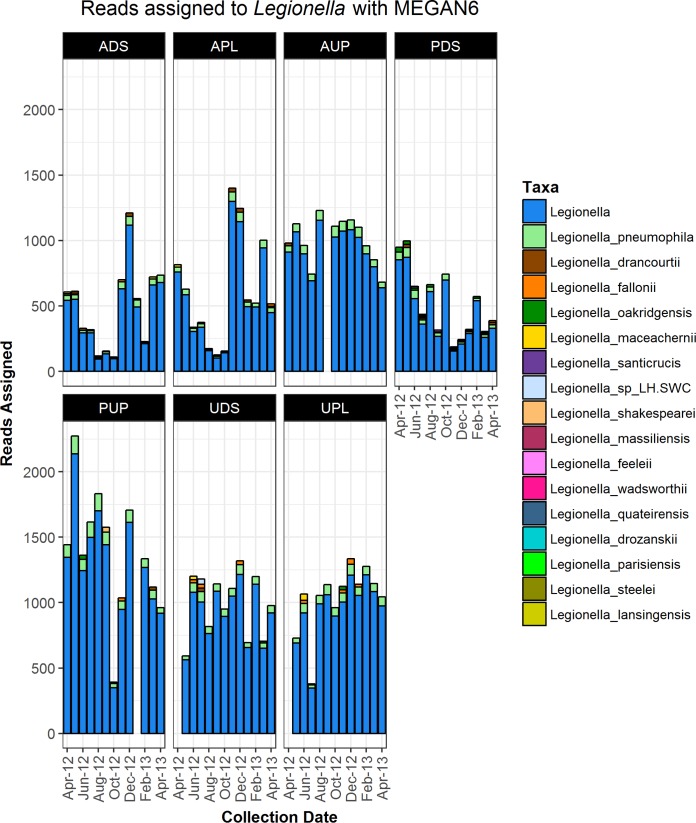
Taxonomic classification of *Legionella* metagenome shotgun sequencing reads. The numbers of metagenomic sequencing reads classified by MEGAN6 analysis to *Legionella* are shown for each sampling site and date of collection. Reads assigned to *Legionella* spp. for which the species-level assignment comprised fewer than 2% of the total reads assigned to *Legionella* are denoted “Legionella” in the legend (i.e., “Legionella” denotes the sum of all reads assigned to the genus *Legionella* and all *Legionella* species not depicted separately). Species in the legend are ordered from most abundant to least abundant in the overall data set (all samples).

To examine the distribution of *Legionella* spp. at a higher resolution than the genus level, the shotgun metagenomics data set was used to obtain species-level classification. The shotgun metagenomics reads were classified to >40 *Legionella* species by using MEGAN6, albeit with very few reads associated with some species (Fig. 2). A majority of sequence reads were assigned only at the *Legionella* genus level. Among those reads identified at the species level, *L. pneumophila* was the most abundant at all sampling sites.

In addition, we used a BLAST approach to identify sequence reads from the shotgun metagenomics data set matching the *mip* gene, which is used extensively for *Legionella* species determinations (19). This analysis also found a low number of reads matching >35 different *Legionella* spp. among the samples examined (see Table S2). Notably, the range of nucleotide identity among the reads aligned with known *mip* genes varied widely (~68 to 94%).

### Diversity of *Legionella* isolates relative to other bacterial taxa.

**FIG 3:**
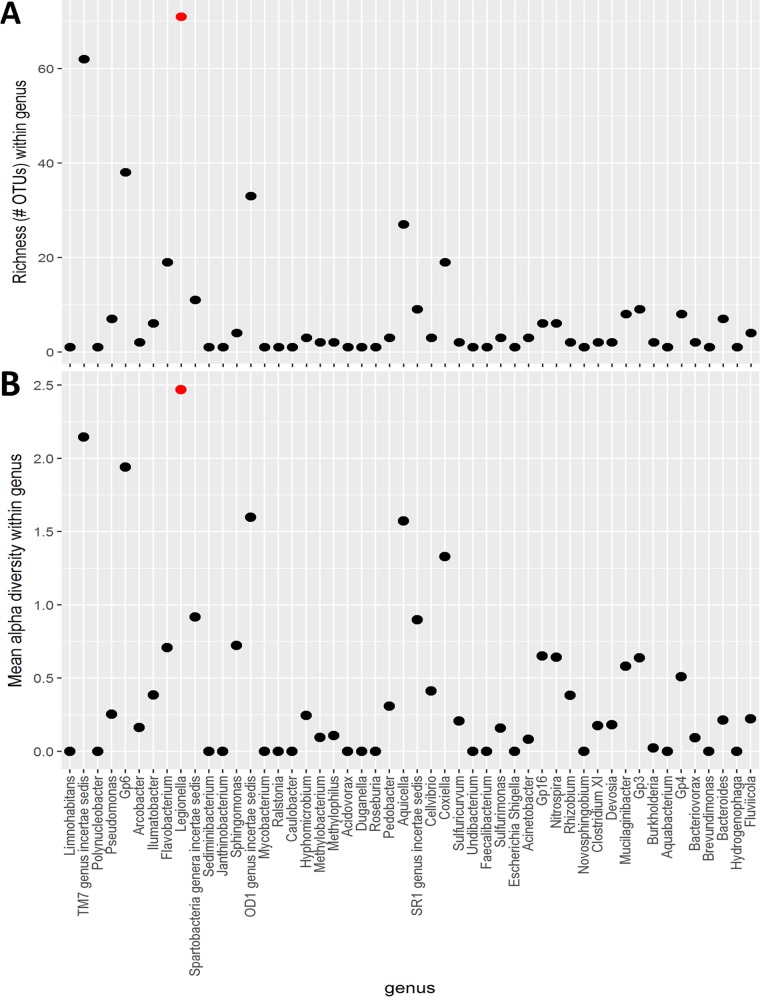
Comparison of diversity levels of selected bacterial taxa. The top 50 most abundant genera derived from 16S rRNA sequencing are shown in order of greatest to least abundance, from left to right. (A) The number of OTUs (richness) of each taxon is shown. (B) Mean alpha diversity was calculated based on the OTUs assigned to each genus shown. Richness and alpha diversity values for *Legionella* are shown by the red dots in each chart.

To better understand the diversity of *Legionella* spp. present in the samples, the 16S rRNA gene data sets were examined at the operational taxonomic unit (OTU) level. OTUs approximate microbial taxa, but are not limited to previously sequenced taxa (as in the MEGAN6 analysis), thus providing a useful complement to the species-level classification performed with metagenomics data. A total of 71 OTUs were assigned to *Legionella*, supporting the view of a highly diverse population of *Legionella* spp. present in these samples (Fig. 3A). *Legionella* is the ninth most abundant genus in the data set, so to rule out the possibility of the richness being driven by relative abundance, the richness of *Legionella* was compared to other abundant genera. The top 50 most abundant genera in the data set over all sampling sites, based on the sum of all reads assigned to the genera from all samples, were plotted versus the richness or number of OTUs assigned to that genus (see Fig. S2 in our figshare files). Richness was unevenly distributed among the taxa and did not seem to be driven by relative abundance. Most genera had a richness of fewer than 10 OTUs, and only 5 genera had a richness greater than 20 OTUs.

*Legionella* had markedly higher richness than the mean (8.1 OTUs) and median (2 OTUs) richness observed among the top 50 most abundant genera. This high richness of *Legionella* was even more pronounced compared to the entire data set (versus just the top 50), which had a mean richness of 3.2 and a median of 1. The richness of OTUs assigned to *Legionella* was distributed across sample sites and time (see Fig. S3). Finally, average alpha diversity values over all samples of the 50 most abundant genera were also plotted and showed that *Legionella* was not only the richest but also the most diverse bacterial genus identified (Fig. 3B).

### Comparison of *Legionella* and *Amoebozoa* relative abundances.

**FIG 4:**
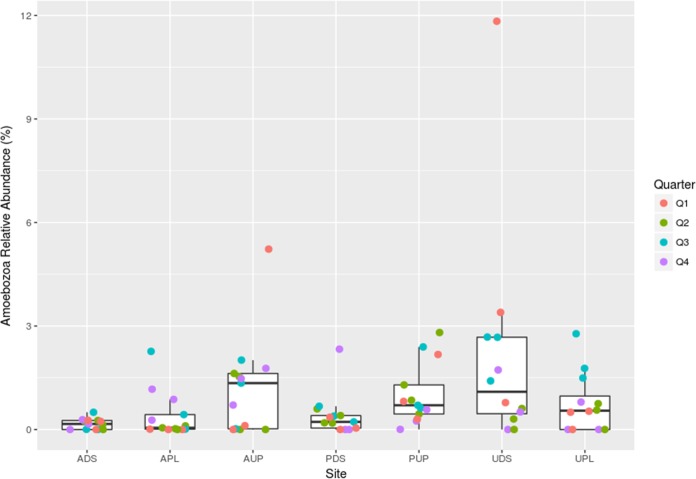
Relative abundance of *Amoebozoa* at various sites. Box plots represent the relative abundances of *Amoebozoa* present for all samples obtained from the indicated site, determined by 18S rRNA sequencing. Each circle represents the relative abundance of *Amoebozoa* in a specific sample. Circles are color coded by the date of sample collection, where quarters of the year are designated as follows: Q1, January, February, and March; Q2, April, May, and June; Q3, July, August, and September; Q4, October, November, and December. Boxes are bounded by the 25th and 75th percentiles, and the middle line represents the median relative abundance.

Analysis of the 18S rRNA gene data set revealed a wide distribution in the relative abundance levels of the phylum *Amoebozoa* among the sample sites (Fig. 4). A significant difference (*q-*value, <0.05) was observed between the site downstream of agricultural activity (ADS) and the PUP and urban downstream (UDS) sites (see Table S3). The more pristine sites within the AUP and PUP watersheds displayed wider distributions of *Amoebozoa* relative abundance levels than the more impacted sites (ADS and PDS [the site downstream from the protected reservoir]). The most pronounced seasonal effect was observed in the urban floodplain (UPL) site, where samples collected between July and September had the highest relative abundance of *Amoebozoa*. (See Materials and Methods for further descriptions of the sampling sites.)

*Platyamoeba* was the most abundant genus (mean relative abundance over all samples, ~0.3%) represented among the *Amoebozoa* (data not shown). *Acanthamoeba*, a well-known host of *Legionella*, had a mean relative abundance that was nearly 100-fold lower than that of *Platyamoeba*. *Naegleria*, an amoebal member of a separate phylum which can also host *Legionella*, was not detected in any samples.

## DISCUSSION

The robust methodology described by Uyaguari-Diaz et al. (17) to separate various microbial components (eukaryotic, bacterial, and viral) in natural water samples via both amplicon (16S and 18S) as well as metagenomic sequencing was used to characterize the compositions of water samples from various watersheds in British Columbia. The predominant bacterial phyla in the analysis of seven watershed sampling sites using metagenomic sequencing were *Proteobacteria*, *Actinobacteria*, *Firmicutes*, and *Bacteroidetes*. A year-long study that examined the same sites showed a shift in the microbial community composition (revealed by the average genome composition and k-mer composition) among some of the sites corresponding to the season and/or nutrient concentrations (18). In the current study, we detected *Legionella*, a member of the *Gammaproteobacteria* order, at all sampling sites and collection dates.

Unlike the seasonal patterns observed when examining the composition of the entire bacterial community (18), the relative abundance of *Legionella* varied throughout the year, without any discernible seasonal patterns, reaching as high as 2% of the bacterial taxa present. Although LD cases peak in summer and autumn months, natural waters are not considered a source of disease to the extent that water systems in built environments are considered the sources (1). Other researchers have detected *Legionella* by using culture or quantitative PCR in natural water sources. In a recent study, investigators found ~10^4^ to 10^5^ cells/liter of *Legionella* spp. in some Taiwanese river water samples via real-time PCR (20), while a previous study of marine and freshwater sites in Puerto Rico demonstrated an abundance of *L. pneumophila* of 10^4^ cells/ml via direct fluorescence antibody (DFA) testing (21). These findings suggest that the quantity of *Legionella* in the natural environment may be highly variable.

The relative abundance of *Legionella* was highest in sites with limited land use: from the site upstream of agricultural activity (AUP) and from a river that empties into a drinking water reservoir (PUP). In both cases, these sources feed downstream sites where the relative abundance of *Legionella* is lower. There are several possible explanations for this decrease in *Legionella* relative abundance. Downstream sites may contain contaminants or lack specific nutrients for *Legionella* growth. There may also be an increase in certain non-*Legionella* genera in these downstream sites, resulting in a lower relative abundance of *Legionella* in the community. Alternatively, *Legionella* may become associated with biofilms, which would decrease their relative abundance in the surface water samples collected in this study. Notably, the water collected at the PDS site travels through a nearly 9-km pipe made of concrete and steel and lined with coal tar. It is possible that biofilms present within this pipe may trap *Legionella*; *Legionella* can also survive and grow within various amoeba species. Similar to the pattern observed with *Legionella* relative abundances, the lowest mean relative abundances of *Amoebozoa* were found at the ADS, APL, and PDS sites, supporting the possibility that *Legionella* relative abundance is amplified by the presence of amoeba in these natural water sources. The differences in relative abundance of *Legionella* seen in our study may also be due to the presence of other protozoa; in addition to amoebas, *L. pneumophila* has been shown to infect and grow within ciliates (22), and protozoan predators have recently been isolated that graze on virulent *Legionella* spp. (23).

Note that the abundance values for both bacteria and eukaryotes were relative rather than absolute abundance values. Furthermore, the relative abundance of eukaryotes inferred by 18S rRNA gene sequencing would be affected by the large variation in copy numbers of the 18S rRNA gene, which can vary by several orders of magnitude between species (24).

This study revealed that *Legionella* spp. present in the watersheds examined are incredibly diverse. More than 70 OTUs were detected via 16S amplicon sequencing. The metagenomic sequence analysis used in this study demonstrated that *L. pneumophila* was the most common species represented. Similarly, Fliermans et al. detected *L. pneumophila* by DFA in nearly all concentrated water samples collected from 67 natural water sources in North Carolina, South Carolina, Georgia, Florida, Alabama, Indiana, and Illinois (10). Various *Legionella* spp. are frequently detected in studies of natural water sources (20, 21, 25, 26). Notably, sequence analysis of the most common *Legionella* 16S rRNA gene-based OTU amplified from water samples along a French river were associated with unknown/uncultured bacteria (25). The high diversity of *Legionella* spp. among these sources may have implications for clinical disease, since several non-*pneumophila Legionella* species are associated with clinical disease (including pneumonia), especially among immunosuppressed populations (2). A study of natural water sources in the Mount St. Helens (Washington, USA) blast zone was conducted after researchers exposed to lakes and streams in the region reported symptoms consistent with Pontiac fever in the early 1980s (27). Various known *Legionella* spp. were detected in this study, with higher organism relative abundances found in water samples taken within the blast zone and in lakes receiving water from hydrothermal seeps than in sites outside the blast zone. A novel species (*L. sainthelensi*) was isolated from water samples collected around Mount St. Helens (28), and this species was subsequently found to be associated with clinical disease (29). Although we did not attempt to isolate and grow the putative novel *Legionella* species from our samples, doing so could be the next step for future studies.

Metagenomics classification programs such as MEGAN6 used in this study may overclassify reads to incorrect species if the matching species is not present in the database (30). More specifically, the program might assign reads to the most closely related species in the database. There are currently over 500 *L. pneumophila* genome sequences in the NCBI database, but only a few representatives are present for other *Legionella* species. The wide range of alignment identities observed with the MEGAN6 analysis further suggests that novel or uncharacterized *Legionella* strains may be present in the samples. Notably, alignment of the shotgun metagenomic reads with the *Legionella mip* gene also uncovered a large number of *Legionella* species (>35) among the watershed samples, but sequencing coverage of this gene may be limited. Nonetheless, the alignment identity of these matches was low (typically <80%), further suggesting the presence of additional novel *Legionella* species in these watersheds.

While the presence of *Legionella* spp. in natural water samples alone is not a significant public health concern, these organisms may seed human-made water systems. In turn, these systems could become sources of *Legionella* dissemination under permissive conditions. Understanding the diversity of organisms present in the natural aquatic environment and factors that may contribute to increased abundance of specific *Legionella* spp. in these environments may help public health workers identify potential new threats to human health and respond quickly to LD by using improved diagnostic and typing assays. This study demonstrates that natural aquatic environments, including watersheds, likely harbor previously unrecognized *Legionella* spp. As culture-independent diagnostic tests for LD become more commonly utilized, it will be important to evaluate the ability of these assays to detect new and emerging *Legionella* spp. and assess their potential to cause disease.

## MATERIALS AND METHODS

### Environmental sampling sites and processing.

Water sample collection and processing have been described previously (17). Water samples were collected monthly from seven sites in three watersheds in southwestern British Columbia, Canada. Contextual data for the samples can be found on the figshare website (https://figshare.com/articles/Sample_data_MIxS_format/5188063). The watersheds had varied land uses: an agricultural watershed, an urban watershed, and a protected watershed that was used as a drinking water source. Three sites within the agricultural watershed were sampled, including a site upstream of agricultural activity (AUP), within a highly farmed and irrigated floodplain (APL), and a site downstream of this activity (ADS). Two sites were sampled in the protected and urban watersheds. A forested and protected river site that empties into a drinking water reservoir (PUP) was sampled, along with a site where water from the reservoir empties out of a 9-km-long pipe (PDS). Finally, urban sites selected from a stream passing through 300 m (UPL) and 1 km downstream (UDS) in a residential development were sampled. Samples were collected between April 2012 and April 2013 for the protected (PUP and PDS) and agriculturally affected (AUP, APL, and ADS) samples. The urban affected samples (UPL and UDS) were collected from May 2012 to April 2013.

For each sample, 40 liters of water was collected and filtered in the field through a 105-μm-pore-size Spectra/mesh polypropylene filter to remove larger particles and debris. Samples were kept on ice and transported to the British Columbia Centre for Disease Control Public Health Laboratory (BCCDC PHL) and were processed within 24 h. Samples were serially filtered to generate size-specific fractions relating to microbial class. A 1-μm-pore-size filter (Envirochek HV; Pall Corporation, Ann Arbor, MI) was used to capture eukaryote-sized particles, followed by a 0.2-μm, 142-mm Supor-200 membrane disk filter (Pall Corporation, Ann Arbor, MI) to capture bacterial and archaeal-sized particles. Eukaryotic-sized cells retained in the 1-μm Envirochek HV capsules were eluted per the manufacturer’s protocol. For this fraction, before extraction and to facilitate disruption of eukaryotic cells, eight freeze-thaw cycles were followed by digestion overnight with proteinase K (Qiagen Sciences, Germantown, MD). DNA from both the eukaryote- and bacteria-sized fractions were extracted using the PowerLyzer Powersoil DNA isolation kit (MoBio, Inc., Carlsbad, CA), which uses a combination of bead beating and chemical lysis. Particle-associated bacterial cells would have been removed by the 105-μm and 1-μm filters, and so the microbial community collected from the 0.2-μm filter was primarily composed of the free-living bacterio-plankton community. This filtering thus misses microorganisms that are particle associated or are filtered out due to being larger than the filter sizes used.

### Shotgun and amplicon sequencing.

Both shotgun and amplicon sequencing were performed with the Illumina MiSeq (Illumina, Inc., San Diego, CA) using the MiSeq reagent kit V2 (two 250-bp paired-end reads, 500 cycles) at the BCCDC PHL. Each sequence run included a positive control (mock community) and negative control (ultrapure water) sample, as described previously (17). Amplicons were generated as previously described, targeting the V1 to V3 regions of the 18S rRNA gene for the eukaryote-sized fraction and targeting V3 and V4 regions of the 16S rRNA gene for the bacteria-sized fraction (17). Amplicons were purified using the QIAQuick PCR purification kit (Qiagen Sciences, Maryland, MD), and sequencing libraries were prepared using the NEXTflex ChIP-Seq kit (BIOO Scientific, Austin, TX) using the gel size selection option, both per the manufacturer’s instructions. The shotgun sequencing libraries were prepared using the Nextera XT DNA sample preparation kit (Illumina, Inc., San Diego, CA), and gel-size selection was performed using the Ranger technology (Coastal Genomics Inc., Burnaby, BC, Canada) and targeting fragments between 500 and 800 bp (31).

### Bioinformatic analysis.

Quality control for the 16S sequences was performed using Cutadapt (32) and Trimmomatic (33) with default parameters and removing reads from the data set if, following quality filtering and trimming, either read in the pair was less than 200 bp. Following this processing, samples were subsampled to 10,000 reads. Samples that had fewer than 10,000 reads were removed from further analysis (the omitted samples included the May 2012 samples from the ADS, PDS, and UDS sites, the September 2012 sample from the PUP site, and the December 2012 sample from the UDS site). The MiSeq protocol in Mothur was used to generate OTUs and their taxonomic assignments (34, 35). The data were exported in BIOM format (36), and the OTUs were extracted that were assigned to *Legionella*.

Shotgun sequencing reads were first trimmed to remove low-quality bases by using Trimmomatic (33). Trimming at the 3′ end was performed with a sliding window of 5 bases and a minimum Phred score of 20, and trimming at the 5′ end was performed with consecutive bases with Phred scores of less than 20 removed. Sequencing adapters were then removed using CutAdapt (32), overlapping paired-end reads were merged with PEAR (37), and reads shorter than 100 bp were discarded. To normalize the samples, all samples were subsampled to 418,500 reads, the smallest number of reads in a sample (sample 32). The January 2013 sample from the PUP site was omitted from analyses due to too few reads (sample 82). Sequence reads were then run on Diamond version 0.8.36 (38) with default parameters against the NCBI nr database (downloaded 21 February 2017). These results were used as input for taxonomic classification by MN version 6.6.7 (39) using default parameters, except that the Min Support percentage which was set to 0. The taxonomic classification files were then parsed to extract the assignments directly to *Legionella* at the genus level or to any specific *Legionella* species. Additionally, a custom BLAST database containing >700 *Legionella* spp. *mip* nucleotide sequences was used to identify raw reads aligning to at least 100 nucleotides and an *E* value of 1 × 10^−10^ with this gene. BLAST using the NCBI nt database was also performed with the identified reads, and those with better alignments to non-*Legionella* targets were excluded.

Analyses were performed in R (v3.2.3), and Shannon’s diversity index was calculated using the vegan package (40). The Kruskal-Wallis method with Dunn’s test was used to compare the relative abundance of legionellae among the sites, with *P* values adjusted using the Benjamini-Hochberg method. The September 2012 sample from the AUP site was removed from analyses, as it had been previously noted as unusual and perhaps mislabeled during sample processing (18).

Paired-end 18S read files were quality filtered and trimmed with Trim Galore v0.3.7 (available at: http://www.bioinformatics.babraham.ac.uk/projects/trim_galore/) by using a quality score cutoff of 25. Remaining read pairs with more than 10 overlapping nucleotides were joined using FLASH v1.2.11 (41). The merged read pairs were used as input to QIIME v1.9.1 (42), with the SILVA (release 128) 18S database (43) used for chimera filtering and open reference OTU assignment.

### Accession number(s).

Sequence data were submitted to the NCBI Sequence Read Archive under BioProject ID 287840.
